# Talar Body Fracture With Closed Pan-Talar Dislocation

**DOI:** 10.7759/cureus.15349

**Published:** 2021-05-31

**Authors:** Saroj K Patra, Shakti Swaroop, Bishnu P Patro, Sashikant Panda, Ritesh Panda

**Affiliations:** 1 Trauma & Orthopaedics, All India Institute of Medical Sciences, Bhubaneswar, IND; 2 Orthopaedics, All India Institute of Medical Sciences, Bhubaneswar, IND; 3 Orthopaedics, All India Institute of Medical Sciences, BHUBANESWAR, IND; 4 Plastic Surgery, All India Institute of Medical Sciences, Bhubaneswar, IND

**Keywords:** talus, body, fracture, pan-talar dislocation, ankle injury

## Abstract

Talar body fracture associated with pan-talar (tibiotalar, talocalcaneal, talonavicular) dislocation is a rare condition. Timely intervention with anatomical reduction will lead to better healing of the fracture. It will result in articular congruity of the talus and less chance of secondary arthritis. We describe a rare talar body fracture with pan-talar dislocation, which does not fit into any of the classifications mentioned in the literature.

A young male of 21 years old was admitted to the trauma and emergency department of a tertiary care hospital within three hours of injury. Following the radiological investigations, the patient was found to have a talar body fracture with pan-talar dislocation. Initially, a close reduction was attempted which failed. Subsequently, it was managed with open reduction and internal fixation after eight hours of injury. Talar body fracture was fixed with cannulated cancellous screws and ankle stabilized with joint spanning external fixator. At six months the patient had satisfactory healing of fracture without any irregularity of the articular surface of talus or arthritic changes of involved joints.

Early surgical intervention with anatomical reduction of talar body fracture with pan-talar dislocation may lead to better union and less chance of secondary arthritis of peri talar joints.

## Introduction

Fractures of the talus bone are not common and contribute 0.1 to 0.85 % of all fractures [1,2]. Of all fractures of the talus; talar body fractures account for 37%, neck fractures account for 50%, and head fractures account for 13% [3]. Talar body fractures usually occur with high-velocity trauma and, more commonly associated with medial malleolus fracture [4]. Talus fracture is associated with high morbidity due to osteonecrosis, subtalar arthritis, ankle joint arthritis, and surrounding soft tissue complications. Literature regarding talar fractures is limited to few case series and specific case reports. We describe a case of atypical fracture-dislocation of the body of talus, which does not fit into Sneppen’s classification, Delee’s classification, and Orthopaedic Trauma Association (OTA) classification.

## Case presentation

A 21-year-old male presented to a tertiary care hospital’s trauma and emergency department following a road traffic accident within three hours of injury. At the time of trauma, the foot was forced into eversion and dorsiflexion. He complained of pain in the left ankle (9/10 visual analog score) with loss of ankle congruity and inability to bear weight.

**Figure 1:**
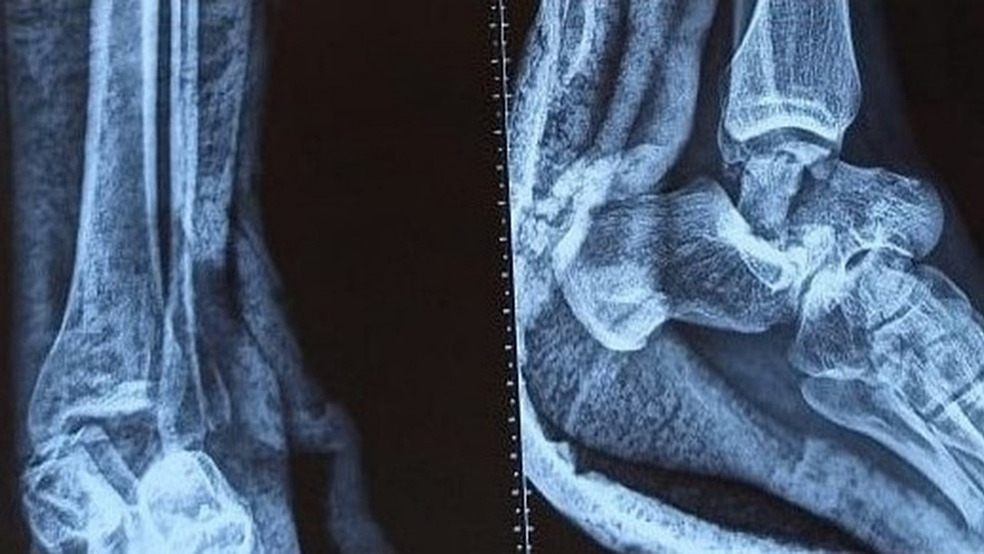
Preoperative X-ray

His vitals were stable, and the left ankle was swollen with tenting of the skin over the medial aspect along with skin discoloration. The distal neurovascular status of the affected limb was normal. The left ankle was immobilized in a below-knee splint after stabilizing the patient. Radiographs of the ankle and foot, along with trauma series radiographs, were taken. The radiograph showed a fracture-dislocation of the talar body and fracture of the lateral malleolus tip (Figure 1).

**Figure 2:**
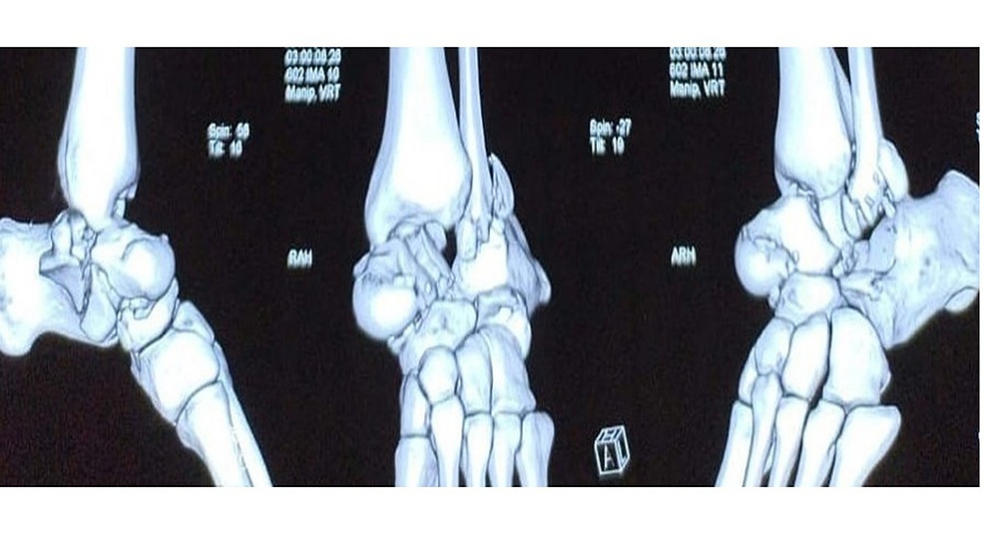
Preoperative CT scan showing Pan-talar dislocation

CT scan showed a fracture of the talar body with pan-talar (tibiotalar, talocalcaneal, talonavicular) dislocation (Figure 2).

Closed reduction was attempted under sedation but failed, so next, the patient was prepared for emergency surgery.

**Figure 3:**
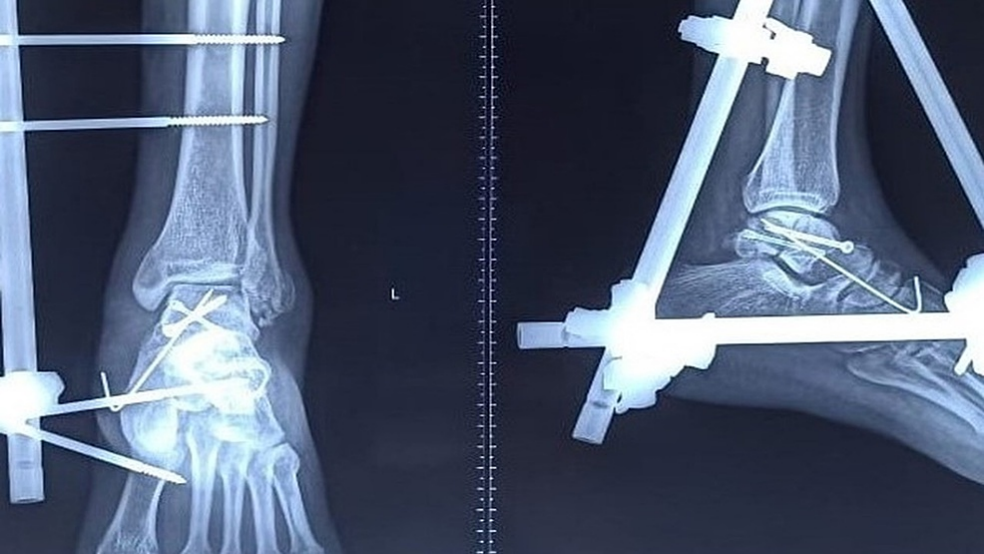
Postoperative X-ray showing fixation of talus with external fixator

Talus fracture was exposed by the direct medial approach over the head of the talus as it was void of soft tissues and was directly under the skin. The tibialis anterior tendon was preventing reduction of the talar head. After retracting the tendon the body of the talus reduced and fixed with four mm partially threaded cannulated screws anteromedial to the posterolateral aspect of the body under fluoroscopy. After the reconstruction of the talus, the joints were reduced in sequence, first the tibiotalar, second the talocalcaneal, and finally the talonavicular joint and further temporarily fixed with K wires. Another headless cannulated screw was introduced percutaneously from the posteromedial to the body’s anterolateral aspect to achieve further stability. The lateral malleolar fracture was fixed with a K-wire. Fracture reduction and implant position was verified under fluoroscopy (Figure 3).

**Figure 4:**
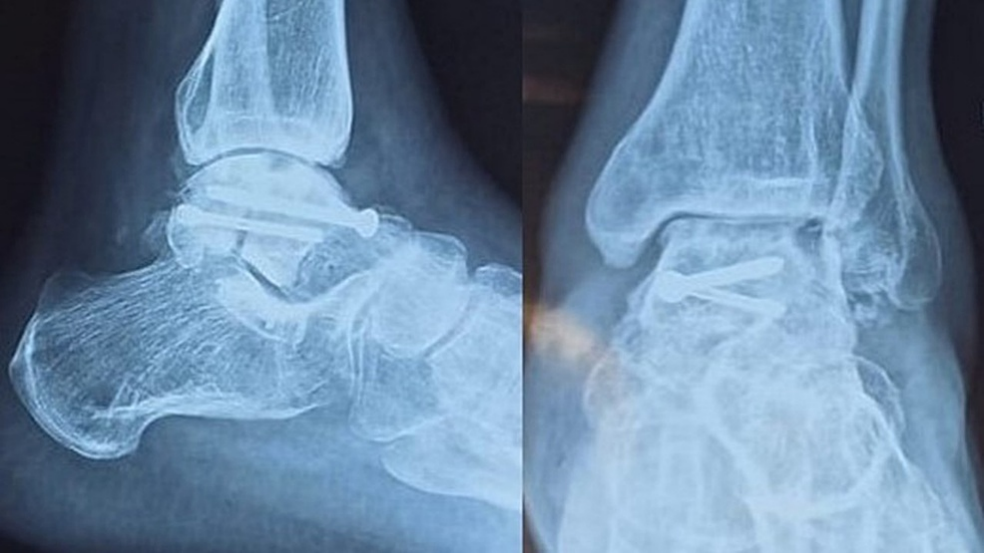
Postoperative X-ray at six month showing lucency at subchondral zone (positive Hawkin's sign)

An external fixator was put to stabilize the ankle joint. The deltoid ligament was disrupted entirely, and a repair was not possible. The skin condition started improving after the third postoperative day, and the patient was discharged home after five days of hospital stay. K-wires and external fixators were removed after six weeks with a below-knee pop slab. He was allowed assisted weight-bearing after three months with a PTB (Patella tendon bearing) cast. His post-operative radiograph did not show any signs of avascular necrosis, however there is some lucency at lateral subchondral region which signifies intact vascularity (positive Hawkins's sign) (Figure 4).

**Figure 5:**
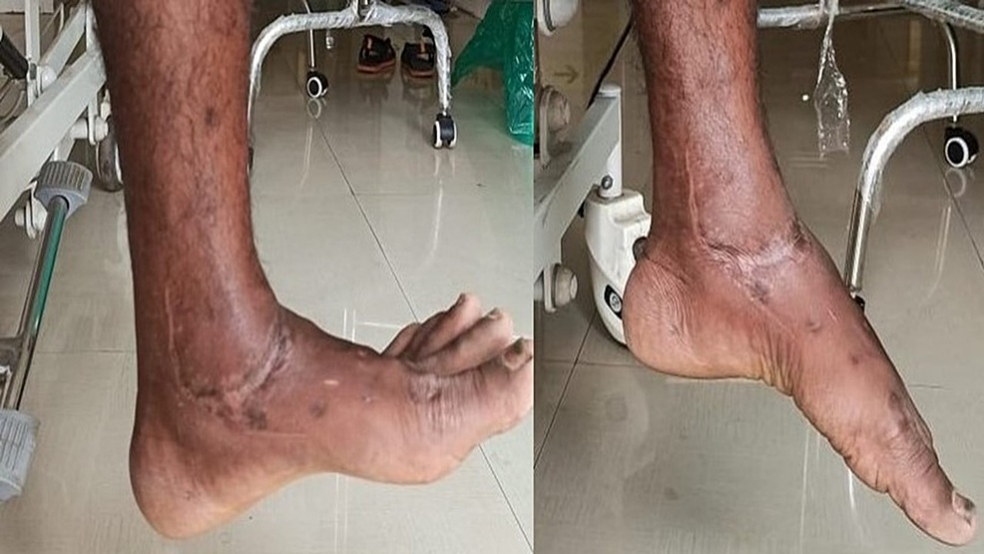
Movement of ankle joint at six month

Fracture united well without any articular incongruity. At six months patient had 20 degrees of active dorsiflexion, 40 degrees of active plantarflexion, 10 degrees of active eversion and inversion (Figure 5).

There is no sclerosis of talar dome in x-ray signifying AVN (Avascular Necrosis) changes. 

## Discussion

Talus is the second most commonly fractured tarsal bone. Violent force is needed for talar fracture to occur. When the fracture line is posterior to the lateral process in the radiograph, it is called talar body fracture. There have been various classifications for the talar body fracture, but none have described this injury pattern. Atypical cases of talar body fractures have been reported previously by Hafez et al., Clement et al., Galanopoulos et al., and Stirton et al. Still, this case is different from all of them and hence needs a mention in the literature. An emergency reduction is essential to prevent skin necrosis and osteonecrosis in the future. The blood supply to the talus’ body is primarily through the tarsal canal’s artery, which supplies the middle one-half to two-thirds directly and through anastomosis potentially supplies the entire body. Its largest branches enter through the inferior aspect of the neck of the talus. The chances of osteonecrosis are as high as 50% percent, but rates of non-union are rare [5]. Post-traumatic arthrosis is common as the talar body fracture involves both the tibiotalar and subtalar joint surfaces and bears the body’s whole weight over a small surface area. Therefore, the recommended management is open reduction and internal fixation with cannulated cancellous screws. Closed reduction and cast application or percutaneous screws application may be preferred for un-displaced or minimally displaced fractures. The type of approach with medial malleolar osteotomy or lateral fibular door osteotomy is recommended to increase the talar body’s visualization depending on the pattern of displacement and comminution of the fracture.

However, it is essential to respect the soft tissue cover, most notably the deltoid ligament, a vital blood supply source to the talar body. In our case, we did not need a medial malleolus osteotomy as the medial deltoid ligament was disrupted, and tibiotalar, subtalar, and talo-navicular joints were dislocated resulting in exposed head and neck with part of the talar body.

The reported rates of malunion are low as compared to post-traumatic arthrosis in talar body fracture. The worse prognosis was associated with comminution, open injuries, and concurrent talar neck fractures [6]. This case is a rare pattern of injury, and it does not fit into the various classifications described by Sneppen, Delee, or the OTA classification [7]. Our management method has yielded promising results, and we wish to add this to the present literature of atypical talar body fractures.

## Conclusions

Fracture dislocation of talus can present in many ways as per the mode of injury. Some pattern of injury may be new as per literature. But early surgical intervention and anatomical reduction give satisfactory results without avascular necrosis and residual arthritis.
